# The Impact of Membrane Lipid Composition on Macrophage Activation in the Immune Defense against *Rhodococcus equi* and *Pseudomonas aeruginosa*

**DOI:** 10.3390/ijms12117510

**Published:** 2011-11-02

**Authors:** Axel Schoeniger, Stephanie Adolph, Herbert Fuhrmann, Julia Schumann

**Affiliations:** Institute of Physiological Chemistry, Faculty of Veterinary Medicine, University of Leipzig, An den Tierkliniken 1, 04103 Leipzig, Germany; E-Mails: axel.schoeniger@vmf.uni-leipzig.de (A.S.); adolph@vetmed.uni-leipzig.de (S.A.); fuhrmann@vmf.uni-leipzig.de (H.F.)

**Keywords:** RAW264.7, macrophage activation, PUFA, IL-1β, IL-6, IL-10, TNF-α, CD86, MyD88, lysozyme

## Abstract

Nutritional fatty acids are known to have an impact on membrane lipid composition of body cells, including cells of the immune system, thus providing a link between dietary fatty acid uptake, inflammation and immunity. In this study we reveal the significance of macrophage membrane lipid composition on gene expression and cytokine synthesis thereby highlighting signal transduction processes, macrophage activation as well as macrophage defense mechanisms. Using RAW264.7 macrophages as a model system, we identified polyunsaturated fatty acids (PUFA) of both the *n*-3 and the *n*-6 family to down-regulate the synthesis of: (*i*) the pro-inflammatory cytokines IL-1β, IL-6 and TNF-α; (*ii*) the co-stimulatory molecule CD86; as well as (*iii*) the antimicrobial polypeptide lysozyme. The action of the fatty acids partially depended on the activation status of the macrophages. It is particularly important to note that the anti-inflammatory action of the PUFA could also be seen in case of infection of RAW264.7 with viable microorganisms of the genera *R. equi* and *P. aeruginosa*. In summary, our data provide strong evidence that PUFA from both the *n*-3 and the *n*-6 family down-regulate inflammation processes in context of chronic infections caused by persistent pathogens.

## 1. Introduction

Fatty acids are an integral part of cellular membranes. The heterogeneity of fatty acids in the membrane contributes to membrane fluidity as well as to the physical and chemical properties of various membrane domains [1,2]. In fact, basic properties of membranes as fatty acid chain order, phase behavior, elastic compressibility, ion permeability, fusion, rapid flip-flop as well as protein function are known to be determined by the lipid composition [1–3]. Accordingly, numerous cellular functions critically rely on the dynamic characteristics of the membranes. This includes cell signaling mechanisms as well as catalytic processes by membrane-associated enzymes [1,2]. Of note, nutritional fatty acids are known to have an impact on membrane lipid composition of body cells, including cells of the immune system [4–7]. Thus, there is a link between dietary fatty acid uptake, inflammation and immunity.

Infectious diseases occur when a microorganism succeeds in overwhelming innate host defenses. Macrophages play a key role in innate immunity due to their ability to detect and destroy pathogens without the help of an adaptive immune response [8,9]. The antigen-presenting scavenger cells serve as a source of cytokines like IL-1β, IL-6, IL-10 and TNF-α, and are crucial for orchestrating the immune response [10,11]. Evading the macrophage defense mechanisms therefore is a precondition for microorganisms to establish a local site of infection, to replicate and to successfully disseminate within the host. Immunocompromised individuals are in special danger of developing serious infections with *Rhodococcus equi* and *Pseudomonas aeruginosa* [12–14].

*R. equi*, a Gram-positive, aerobic, facultative intracellular and immotile soil bacterium, is known as a pulmonary pathogen of young horses and immunocompromised humans, such as AIDS patients [13–15]. There are both virulent and non-virulent isolates. The infectivity depends on the presence of distinct virulence-associated proteins and plasmids. The pathogen has been shown to survive and to multiply in macrophages via arresting the normal pathway of phagosome maturation [16,17]. Once internalized *R. equi* prevents the fusion of its phagosome with lysosomes thus leading to an arrested phagosome neutral in pH and without any lysosomal contents [16,17]. In this way *R. equi* is able to establish a niche for survival inside the host cell [17].

*P. aeruginosa*, a Gram-negative, facultative anaerobic bacterium with unipolar motility, is known as a nosocomial pathogen of immunocompromised individuals [18]. Tissues typically infected are the pulmonary tract, urinary tract, burns and wounds [12]. Moreover, *P. aeruginosa* is reported to be the most frequent colonizer of medical devices (e.g., catheters) [12]. Infections with *P. aeruginosa* are often difficult to treat [19]. The pathogen has been demonstrated to exhibit numerous enzymatic and mutational mechanisms of bacterial resistance [19,20]. Environmental persistence is further increased by the ability of *P. aeruginosa* to form biofilms [21]. In addition, the microorganism has been reported to synchronize gene expression by an intercellular communication mechanism, the quorum sensing [21,22]. This mechanism enables the bacterial population to act as a single organism and to modulate a number of virulence factors, including biofilm formation as well as the production of numerous toxins [21,22].

Feeding studies concerning the impact of PUFA supplementation on immune defense mechanisms yielded conflicting findings, so far. This is aggravated by variations in experimental settings leading to a lack in comparability of gained results. Moreover, virtually no data regarding the relevance of PUFA in case of macrophage infection with *R. equi* or *P. aeruginosa* exist. Hence, in this *in vitro*-study under defined conditions, we reveal the implication of stimulation and PUFA supplementation on key proteins of innate immune response.

## 2. Results and Discussion

### 2.1. Cytokines

Cytokines play a pivotal role in intercellular communication acting as immune-modulating. In particular, macrophages are potent producers of the small cell-signaling protein molecules including the pro-inflammatory IL-1β, IL-6 and TNF-α as well as the anti-inflammatory IL-10. The mediator molecules are a crucial component of host defense. However, pro-inflammatory cytokines also are held responsible for the destruction of host tissue [23].

**Figure 1:**
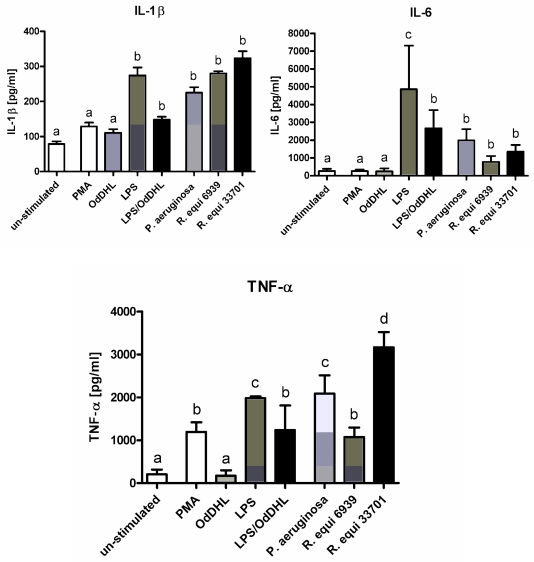
Concentration of IL-1β, IL-6 and TNF-α in supernatants of RAW264.7 macrophages, cultured in basic medium after 24 h of stimulation with lipopolysaccharide (LPS), *N*-(3-oxododecanoyl)-l-homoserine lactone (OdDHL), LPS + OdDHL, phorbol-12-myristate-13-acetate (PMA), *P. aeruginosa* ATCC 10145, *R. equi* ATCC 6939 and *R. equi* ATCC 33701 respectively. Data are mean ± SD (*n* = 6). Bars denoted by different letters are significantly different.

Stimulation of RAW264.7 with lipopolysaccharide (LPS), phorbol-12-myristate-13-acetate (PMA), *P. aeruginosa* and *R. equi* respectively induced an increase in the concentration of pro-inflammatory cytokines in cell supernatants (Figure 1). Significant differences depending on the stimulator added could be assessed. Treatment of the cells with LPS resulted in a significant increase in the concentration of IL-1β, IL-6 as well as TNF-α (Figure 1). In contrast, after stimulation of the macrophages with PMA a significant increase could only be seen for TNF-α (Figure 1). Addition of the quorum sensing molecule N3-oxododecanoyl-l-homoserine lactone (OdDHL) to the culture medium did not affect the concentration of pro-inflammatory cytokines in cell supernatants (Figure 1). The combination of LPS and OdDHL abrogated the stimulating effect of LPS on IL-1β, IL-6 and TNF-α synthesis (Figure 1). Culturing of RAW264.7 in presence of the viable pathogens *P. aeruginosa* and *R. equi* boosted proinflammatory cytokine synthesis as well (Figure 1). The virulent strain *R. equi* ATCC 33701 was found to act more effectively in increasing the production of IL-1β, IL-6 and TNF-α by the macrophages than the non-virulent strain *R. equi* ATCC 6939 (Figure 1).

**Figure 2:**
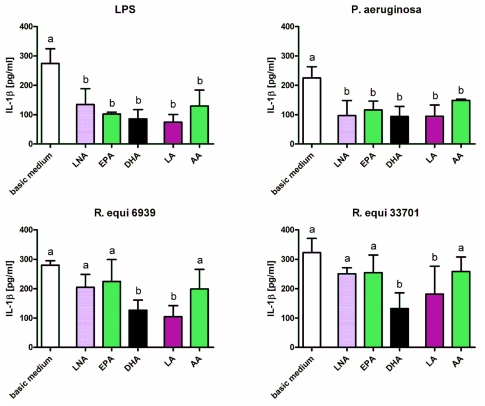
Concentration of IL-1β in supernatants of RAW264.7 macrophages cultured in basic medium as well as in medium supplemented with 15 μmol/L alpha-linolenic acid (LNA), eicosapentaenoic acid (EPA), docosahexaenoic acid (DHA), linoleic acid (LA) or arachidonic acid (AA) after 24 h of stimulation with LPS, *P. aeruginosa* ATCC 10145, *R. equi* ATCC 6939 and *R. equi* ATCC 33701 respectively. Data are mean ± SD (*n* = 6). Bars denoted by different letters are significantly different.

**Figure 3:**
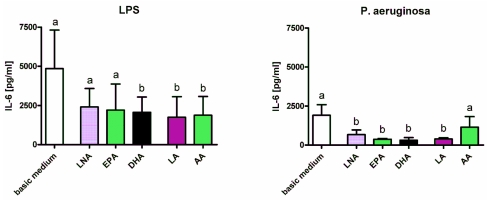

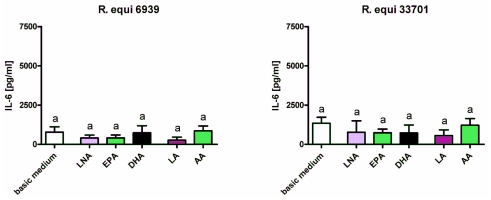
Concentration of IL-6 in supernatants of RAW264.7 macrophages cultured in basic medium as well as in medium supplemented with 15 μmol/L alpha-linolenic acid (LNA), eicosapentaenoic acid (EPA), docosahexaenoic acid (DHA), linoleic acid (LA) or arachidonic acid (AA) after 24 h of stimulation with LPS, *P. aeruginosa* ATCC 10145, *R. equi* ATCC 6939 and *R. equi* ATCC 33701 respectively. Data are mean ± SD (*n* = 6). Bars denoted by different letters are significantly different.

**Figure 4:**
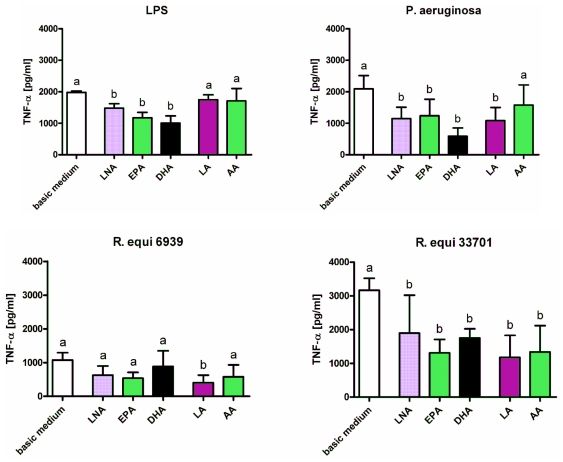
Concentration of TNF-α in supernatants of RAW264.7 macrophages cultured in basic medium as well as in medium supplemented with 15 μmol/L alpha-linolenic acid (LNA), eicosapentaenoic acid (EPA), docosahexaenoic acid (DHA), linoleic acid (LA) or arachidonic acid (AA) after 24 h of stimulation with LPS, *P. aeruginosa* ATCC 10145, *R. equi* ATCC 6939 and *R. equi* ATCC 33701 respectively. Data are mean ± SD (*n* = 6). Bars denoted by different letters are significantly different.

Enrichment of the culture medium with fatty acids diminished the stimulatory effects of LPS, *P. aeruginosa* and *R. equi*. Concentration of IL-1β in supernatants of RAW264.7 stimulated with LPS or *P. aeruginosa* was decreased significantly following feeding of cells with the *n*-3 PUFA alpha-linolenic acid (LNA), eicosapentaenoic acid (EPA) and docosahexaenoic acid (DHA) as well as the *n*-6 PUFA linoleic acid (LA) and arachidonic acid (AA) (Figure 2). In context of *R. equi*-stimulation a significant reduction in IL-1β concentration could only be seen for macrophages supplemented with DHA and LA regardless the *R. equi*-strain tested (Figure 2). Concentration of IL-6 in supernatants of RAW264.7 was decreased significantly following feeding of cells with DHA, LA or AA for LPS stimulation as well as following feeding of cells with LNA, EPA, DHA or LA for stimulation with *P. aeruginosa* (Figure 3). For RAW264.7 stimulated with *R. equi* ATCC 6939 or *R. equi* ATCC 33701 no effect of PUFA supplementation on IL-6 production was seen (Figure 3). PUFA that had a decreasing effect on the secretion of TNF-α by the macrophages were LNA, EPA and DHA for LPS stimulated cells, LNA, EPA, DHA and LA for cells treated with *P. aeruginosa*, LA for cells treated with the non-virulent strain *R. equi* ATCC 6939 as well as LNA, EPA, DHA, LA and AA for cells treated with the virulent strain *R. equi* ATCC 33701 (Figure 4). For un-stimulated cells as well as for cells treated with PMA no effects of PUFA supplementation on the production of the pro-inflammatory cytokines IL-1β, IL-6 and TNF-α could be seen (data not shown). Furthermore, treatment of the cells with LPS in combination with the quorum sensing molecule OdDHL abolished the PUFA effects observed for LPS stimulated RAW264.7 (data not shown).

**Figure 5:**
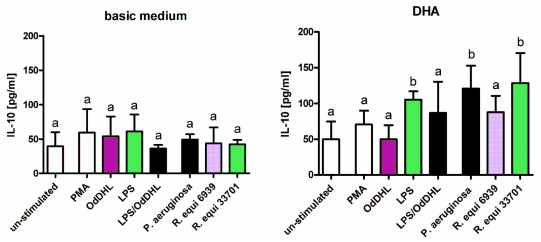
Concentration of IL-10 in supernatants of RAW264.7 macrophages cultured in basic medium as well as in medium supplemented with 15 μmol/L docosahexaenoic acid (DHA) after 24 h of stimulation with LPS, PMA, *P. aeruginosa* ATCC 10145, *R. equi* ATCC 6939 and *R. equi* ATCC 33701 respectively. Data are mean ± SD (*n* = 6). Bars denoted by different letters are significantly different.

Synthesis of the anti-inflammatory cytokine IL-10 was not significantly affected by stimulation of RAW264.7 with LPS, PMA, *P. aeruginosa* and *R. equi* respectively (Figure 5). Likewise, supplementation of the macrophages with the *n*-3 PUFA LNA and EPA or the *n*-6 PUFA LA and AA did not result in any changes in IL-10 concentration in cell supernatants both for un-stimulated as well as for stimulated cells (data not shown). Enrichment of the culture medium with DHA, however, brought about a significant increase in IL-10 production and secretion of RAW264.7 stimulated with LPS, *P. aeruginosa* and *R. equi* respectively (Figure 5). Treatment of the cells with LPS in combination with OdDHL abolished this effect (Figure 5).

In summary, our data underline the interaction of PUFA and cytokine production by activated macrophages. The results presented here demonstrate the fatty acids to arrest the secretion of pro-inflammatory cytokines and, moreover, for DHA to promote the production of an anti-inflammatory cytokine. So far, the inhibitory effect of unsaturated fatty acids on macrophage synthesis of IL-1β, IL-6 and TNF-α has solely been shown for the *n*-3 PUFA EPA and DHA [24,25]. Likewise, to our best knowledge, the effects of PUFA supplementations on macrophage IL-10 synthesis has solely elucidated for these two *n*-3 fatty acids [26]. Thus, our results extend the current knowledge providing evidence that PUFA of both the *n*-3 and the *n*-6 family drive macrophage immune response into an anti-inflammatory direction. Of note, the immune suppressing effect of the fatty acids tested could be seen for adequately stimulated macrophages only. This indicates that the PUFA may interfere with intracellular signal transduction processes related with macrophage activation thus resulting in an inhibited up-regulation of cytokine synthesis.

Macrophages respond to a variety of pathogen-associated molecular patterns (PAMPs) by means of a wide repertoire of distinct pattern recognition receptors (PRRs). LPS is recognized by Toll-like receptor 4 (TLR4) [27,28]. The viable pathogens *R. equi* and *P. aeruginosa*, which are characterized by multiple pathogen-specific PAMPs, are sensed by several TLRs including TLR2, TLR4 and TLR5 [23,27–29]. Following ligand binding TLRs are reported to dimerize and to be recruited into lipid rafts, where they interact with downstream adaptor molecules [30,31]. Data from our previous studies demonstrate that supplementation of immune cells with PUFA results in an incorporation of the fatty acids into cellular membranes, which is accompanied with a modulation of lipid composition in membrane rafts [31]. In fact, inhibition of lipid raft formation by PUFA has already been shown to reduce the recruitment of signaling molecules, such as TLR4, into the rafts [30,31]. PUFA enrichment in membrane rafts therefore seems to be a feasible mechanism of action of the fatty acid-mediated suppression of cytokine synthesis up-regulation by stimulated macrophages.

### 2.2. Surface Molecules

Beside cytokines macrophages also communicate with other cells of the immune system by means of several distinct surface molecules such as the major histocompatibility complex II (MHCII) and the CD antigen 86. MHC class II molecules present antigenic peptides generated in intracellular vesicles of the macrophage to T helper cells. The interaction of a specific peptide:MHC complex with the T cell receptor activates the naive T cell. However, a second signal, maintained via interaction of the co-stimulatory molecule CD86 with CD28, is needed to stimulate the clonal expansion of naive T cells. A further surface molecule expressed by macrophages is the Fc receptor. Fc receptors recognize the Fc portion of immunoglobulins. Ligand binding results in an activation of the macrophage, which is characterized by an increase in the rate of phagocytosis as well as an increase in its bactericidal activity.

**Figure 6:**
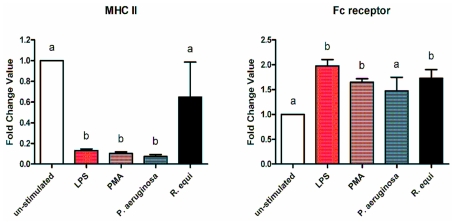
Expression of the MHCII and the Fc receptor mRNA by RAW264.7 macrophages cultured in basic medium after 24 h of stimulation with LPS, PMA, *P. aeruginosa* ATCC 10145 and *R. equi* ATCC 33701 respectively. Data are mean ± SD (*n* = 6). Bars denoted by different letters are significantly different.

Stimulation of RAW264.7 with LPS, PMA, *R. equi* and *P. aeruginosa* respectively resulted in a down-regulation of the MHCII gene expression (Figure 6). For LPS, PMA and *P. aeruginosa* the expression level dropped to a tenth compared to untreated cells (Figure 6). For *R. equi* the abrogating action was less pronounced (Figure 6). No effect of PUFA supplementation on MHCII gene expression could be seen neither for un-stimulated nor for stimulated cells (data not shown).

The expression of the Fc receptor gene was up-regulated by 1.5 to 2 fold following treatment of RAW264.7 with LPS, PMA, *R. equi* and *P. aeruginosa* respectively (Figure 6). There was no effect of PUFA supplementation of cells on the expression level of the Fc receptor gene both for un-stimulated as well as for stimulated RAW264.7 (data not shown).

**Figure 7:**
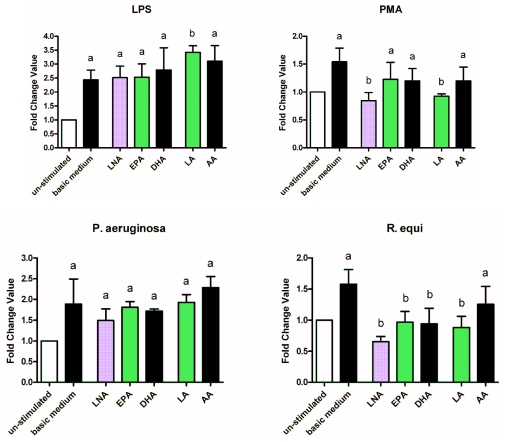
Expression of the CD86 mRNA by RAW264.7 macrophages cultured in basic medium as well as in medium supplemented with 15 μmol/L alpha-linolenic acid (LNA), eicosapentaenoic acid (EPA), docosahexaenoic acid (DHA), linoleic acid (LA) or arachidonic acid (AA) after 24 h of stimulation with LPS, PMA, *P. aeruginosa* ATCC 10145 and *R. equi* ATCC 33701 respectively. Data are mean ± SD (*n* = 6). Bars denoted by different letters are significantly different.

Expression of the CD86 gene increased due to the stimulation of RAW264.7 with LPS, PMA, *R. equi* and *P. aeruginosa* respectively (Figure 7). In the case of PMA and *R. equi* this could be impaired by enrichment of the cells with PUFA. For *R. equi* the fatty acid supplementation completely abrogated the up-regulating effect (Figure 7). Fatty acids identified to act most effective were the *n*-3 PUFA LNA as well as the *n*-6 PUFA LA (Figure 7). Regarding RAW264.7 treated with LPS or *P. aeruginosa* there was no diminishing action of the PUFA added (Figure 7). Likewise, for un-stimulated RAW264.7 no impairment of CD86 gene expression could be seen (data not shown).

**Figure 8:**
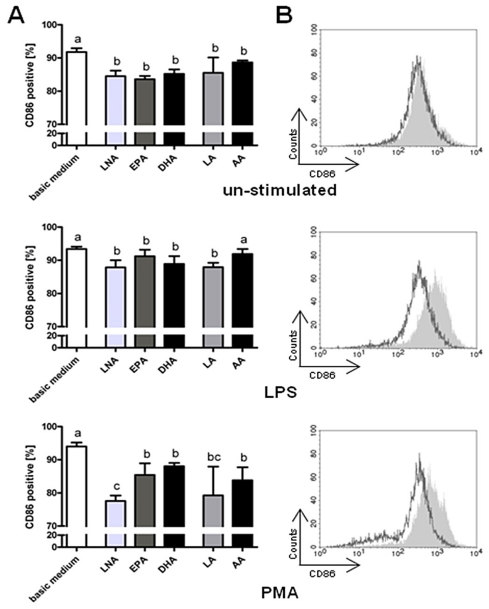

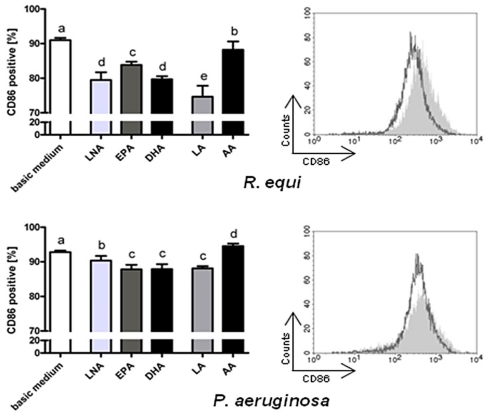
Expression of the surface molecule CD86 by RAW264.7 macrophages cultured in basic medium as well as in medium supplemented with 15 μmol/L alpha-linolenic acid (LNA), eicosapentaenoic acid (EPA), docosahexaenoic acid (DHA), linoleic acid (LA) or arachidonic acid (AA) after 24 h of stimulation with LPS, PMA, *P. aeruginosa* ATCC 10145 and *R. equi* ATCC 33701 respectively. (**A**) Percentage of CD86 positive cells. Data are mean ± SD (*n* = 6). Bars denoted by different letters are significantly different; (**B**) Histograms of surface expression levels of CD86. The grey-shaded histograms correspond to RAW264.7 cultured in basic medium, and empty histograms (dark grey line) correspond to RAW264.7 cultured in LNA supplemented medium. Histograms are representative of one of six experiments.

Flow cytometric analyses confirmed the abrogating effect of a fatty acid enrichment of RAW264.7 on CD86 protein expression. As seen in the gene expression analysis, the diminishing action of the PUFA supplemented was especially apparent for RAW264.7 stimulated with PMA and *R. equi* respectively (Figure 8). Here, too, the *n*-3 PUFA LNA and the *n*-6 PUFA LA were most effective (Figure 8). However, a moderate reduction in CD86 positive cells due to PUFA supplementation could also be seen for RAW264.7 treated with LPS or *P. aeruginosa* as well as for un-stimulated cells (Figure 8).

Taking together, our data demonstrate PUFA to modulate the expression of the co-stimulatory molecule CD86 but not of the MHC class II molecules or the Fc receptor. The diminishing action of unsaturated fatty acids on CD86 expression could be shown for all PUFA tested, in particular LNA and LA. Hence, our data provide first evidence that fatty acids from both the *n*-3 and the *n*-6 family reduce the expression levels of CD86. Of note, the down-regulation of CD86 was demonstrated not only on mRNA level but also on protein level thus underlining the relevance of gained results. Interestingly, on protein level the abrogating effect of the fatty acids added could be seen for both stimulated and un-stimulated RAW264.7. This suggests that there are two independent mechanisms of action. On the one hand PUFA supplementation of cells might modulate membrane raft lipid composition thus interfering with a stimulator-mediated activation of the macrophages, as already discussed. On the other hand PUFA are reported to regulate gene expression via interaction with nuclear and G protein coupled receptors [26,32]. The interaction of the fatty acids with the peroxisome proliferator-activated receptors (PPARs) [33] as well as with the G protein coupled receptor 120 (GPR120) was found to mediate anti-inflammatory effects, particularly in macrophages [32].

### 2.3. Adapter Proteins

Activation of macrophages by PAMPs crucially depends on intracellular adapter proteins, which are accessory to key proteins in signal transduction pathways. The universal adaptor protein used by TLRs is the myeloid differentiation primary response gene 88 (MyD88) [27,28]. Microbial products in the cytoplasm of the cell are sensed by the Nod-like receptors Nod1 and Nod2 [27,28]. The receptor-interacting protein-like interacting caspase-like apoptosis regulatory protein kinase (RICK) functions as the adaptor protein downstream of Nod1 and Nod2 [27,28]. Both, MyD88 and RICK, mediate NFκB and mitogen-activated protein kinase (MAPK) activation in response to specific pathogenic stimuli [27,28].

**Figure 9:**
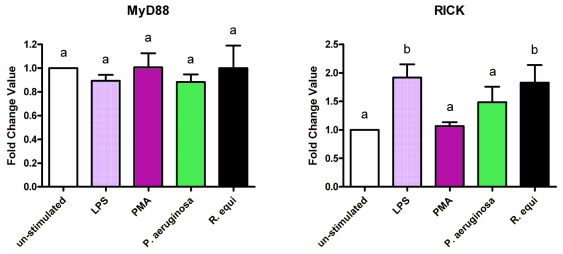
Expression of the MyD88 and the RICK mRNA by RAW264.7 macrophages cultured in basic medium after 24 h of stimulation with LPS, PMA, *P. aeruginosa* ATCC 10145 and *R. equi* ATCC 33701 respectively. Data are mean ± SD (*n* = 6). Bars denoted by different letters are significantly different.

Treatment of RAW264.7 with LPS, PMA, *R. equi* and *P. aeruginosa* respectively did not affect the expression of the MyD88 gene (Figure 9). However, there was a moderate but not significant decrease in MyD88 expression level following enrichment of stimulated cells with PUFA (data not shown). This effect was independent of the kind of stimulator used.

Expression of the RICK gene was up-regulated by stimulation of RAW264.7 with LPS, *R. equi* or *P. aeruginosa* (Figure 9). Treatment of the cells with PMA, in contrast, did not result in any changes in RICK expression level (Figure 9). Enrichment of culture medium with PUFA revealed the fatty acids not to affect the gene expression of RICK. No effect of PUFA supplementation of cells on the expression level of the RICK gene could be seen either for un-stimulated or for stimulated RAW264.7 (data not shown).

Overall, our results support the assumption that the molecular targets of PUFA are signaling components upstream of the adaptor proteins MyD88 and RICK. This is in accordance with previous data reporting that the suppression of NFκB by DHA is not mediated by MyD88 [33].

### 2.4. Lysozyme

Lysozyme, a predominant antimicrobial polypeptide, is present in macrophages in high concentrations. The enzyme exerts bacteriolytic effects due to its peptidoglycane-hydrolyzing activity [34]. Peptidoglycane degradation by lysozyme results in a perturbation of the bacterial cell wall, and, therefore, in the inability of the microorganism to withstand osmotic pressure. Lysozyme can be found in the lysosomes of macrophages, but can also be released in the environment when the cells degranulate providing a first line in innate immune defense [35].

**Figure 10:**
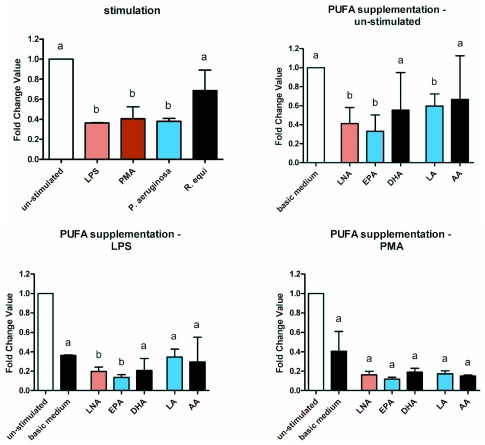

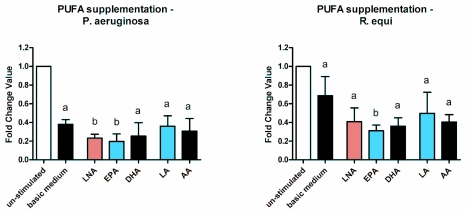
Expression of the lysozyme mRNA by RAW264.7 macrophages cultured in basic medium as well as in medium supplemented with 15 μmol/L alpha-linolenic acid (LNA), eicosapentaenoic acid (EPA), docosahexaenoic acid (DHA), linoleic acid (LA) or arachidonic acid (AA) after 24 h of stimulation with LPS, PMA, *P. aeruginosa* ATCC 10145 and *R. equi* ATCC 33701 respectively. Data are mean ± SD (*n* = 6). Bars denoted by different letters are significantly different.

Treatment of RAW264.7 with LPS, PMA, *R. equi* and *P. aeruginosa* resulted in a marked down-regulation of the lysozyme gene (Figure 10). The expression level dropped by one to two third (Figure 10). Supplementation of the cells with PUFA further decreased lysozyme gene expression (Figure 10). The impairment of the lysozyme gene expression by the fatty acids could be seen for all stimuli tested as well as for un-stimulated RAW264.7 (Figure 10).

**Figure 11:**
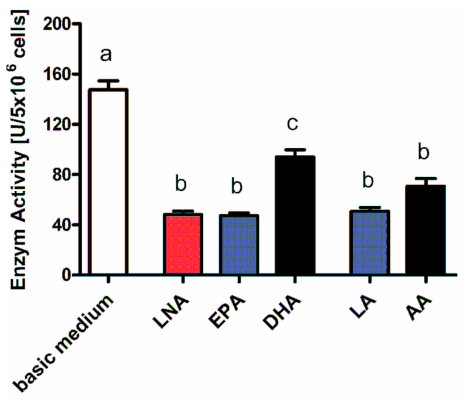
Total enzyme activity of lysozyme of RAW264.7 macrophages cultured in basic medium as well as in medium supplemented with 15 μmol/L alpha-linolenic acid (LNA), eicosapentaenoic acid (EPA), docosahexaenoic acid (DHA), linoleic acid (LA) or arachidonic acid (AA). Data are mean ± SD (*n* = 6). Bars denoted by different letters are significantly different.

Determination of total lysozyme activity confirmed the diminishing effect of the fatty acids. As seen in the gene expression analysis, the abrogating action of the PUFA added emerged both for un-stimulated (Figure 11) and stimulated cells (data not shown).

Summing up, our data clearly demonstrate PUFA to down-regulate lysozyme gene expression resulting in a decrease in enzyme activity. To our knowledge, this is the first time that unsaturated fatty acids were shown to affect lysozyme synthesis. Here, too, however, the immune-suppressive effect of PUFA from both the *n*-3 and the *n*-6 family becomes apparent.

## 3. Experimental Section

### 3.1. Materials

All chemicals and reagents were obtained from Sigma-Aldrich (Taufkirchen, Germany) unless noted otherwise. Cell culture flasks were purchased from Greiner Bio-One (Frickenhausen, Germany). HEPES (25 mmol/L)-buffered RPMI 1640 culture medium containing 300 mg/L l-glutamine was acquired from PAA Laboratories GmbH (Cölbe, Germany).

### 3.2. Cell Culture

The permanent mouse monocyte/macrophage cell line RAW264.7 (ATCC: TIB-71) was used. The RAW264.7 cells were cultured in RPMI 1640 medium supplemented with 4.5 g/L glucose and 5% FCS (basic medium). The fatty acids alpha-linolenic acid (LNA), eicosapentaenoic acid (EPA), docosahexaenoic acid (DHA), linoleic acid (LA) or arachidonic acid (AA) (all Biotrend, Köln, Germany) were included in the culture medium in concentrations of 15 μmol/L using ethanol as a vehicle (0.2% v/v final ethanol concentration). Cells were incubated in 75 cm^2^ cell culture flasks at a density of 1 × 10^6^ cells/mL for 72 h at 37 °C and 5% CO_2_ in a humidified atmosphere. Stimulation of cells was performed for 24 h by means of LPS (1 μg/mL, from *E. coli* serotype 0111:B4), PMA (1 μM), *R. equi* (ATCC: 33701/6939, viable, MOI 0.1), *P. aeruginosa* (ATCC 10145, viable, growth restriction via gentamicin (10 μg/mL), MOI 1), *N*-(3-oxododecanoyl)-l-homoserine lactone (OdDHL, 5 μM) or a combination of LPS (1 μg/mL, from *E. coli* serotype 0111:B4) plus OdDHL (5 μM).

### 3.3. Cytokine Production

RAW264.7 were seeded and stimulated with LPS, OdDHL, LPS + OdDHL, PMA, *P. aeruginosa* ATCC 10145, *R. equi* ATCC 33701 and *R. equi* ATCC 6939 respectively as described above. IL-1β, IL-6, IL-10 and TNF-α were detected in supernatants using murine ELISA kits (Preprotech, London, UK) according to the manufacturer’s instructions. Absorbance was read on a SpectraMax 340PC ELISA reader at 450 nm and analysis done using SoftMax Pro 5 software (all Molecular Device, Munich, Germany). Analysis was performed in triplicates in 6 independent experiments for each combination of fatty acid supplementation and stimulator.

### 3.4. Gene Expression Analysis

RAW264.7 were seeded and stimulated with LPS, PMA, *P. aeruginosa* ATCC 10145 and *R. equi* ATCC 33701 respectively as described above. Gene expression was analyzed by means of a SYBR Green-based quantitative real-time PCR technology. Total RNA was extracted utilizing the RNeasy kit (Qiagen GmbH, Hilden, Germany). Complementary DNA was synthesized using the Oligo(dT)_12–18_Primer and the SuperScript^®^ III Reverse Transcriptase from Invitrogen (Darmstadt, Germany). Quantitative PCR was performed in duplicates using suitable murine RT^2^ qPCR primer assays (SABiosciences, Frederick, USA) and the SensiMix™ SYBR Kit (Bioline, Luckenwalde, Germany). Genes of interest amplified are MYD88, RIPK2, H2-AA, CD86, FCGR2B and LYZ2; housekeeping genes amplified are HPRT and CASC3. Positive controls by means of the XpressRef™ Universal Total RNA (SABiosciences, Frederick, USA) as well as negative controls (*i.e.*, no template control) were prepared for every run. Thermal cycling was carried out on a Rotor-Gene 6000 real time PCR system (Qiagen GmbH, Hilden, Germany) starting with one cycle at 95 °C for 10 min followed by 45 cycles of 95 °C for 15 s, 55 °C for 30 s and 72 °C for 15 s. Relative quantification studies were performed with the Rotor-Gene 6000 Series Software 1.7. The entire procedure was replicated for 3 times on independent RNA isolations.

### 3.5. Lysozyme Assay

RAW264.7 were seeded and stimulated with LPS, PMA, *P. aeruginosa* ATCC 10145 and *R. equi* ATCC 33701 respectively as described above. Total enzyme activity of lysozyme was detected in cell supernatants as well as cell lysates obtained by sonification using the EnzChek^®^ Lysozyme assay kit (Invitrogen, Darmstadt, Germany) according to the manufacturer’s instructions. Fluorescence was read on a SpectraMax 340PC ELISA reader at an excitation wavelength of 485 nm and an emission wavelength of 535 nm, and analysis done using SoftMax Pro 5 software (all Molecular Device, Munich, Germany). Analysis was performed in triplicates in 2 independent experiments for each combination of fatty acid supplementation and stimulator.

### 3.6. CD86 Protein Expression

RAW264.7 were seeded and stimulated with LPS, PMA, *P. aeruginosa* ATCC 10145 and *R. equi* ATCC 33701 respectively as described above. Cells were stained for the surface marker anti-CD86 PE (Miltenyi Biotec GmbH, Bergisch Gladbach, Germany) and fixed in 1% formaldehyde in PBS. In all cases a Fc block (purified anti mouse CD16/CD32) (Miltenyi Biotec GmbH, Bergisch Gladbach, Germany) was used. Specifity of staining was verified via isotope control carried along for the antibody used. Cells were analyzed on a FacsCalibur using Cellquest Pro software (all Becton Dickinson, Heidelberg, Germany). Analysis was performed in triplicates in 2 independent experiments for each combination of fatty acid supplementation and stimulator.

### 3.7. Statistical Analysis

Data are shown as means ± standard deviation (SD). One-way analysis of variance and one-way ANOVA followed by unpaired Students *t* test was used to identify significant differences between means. The statistical analysis was carried out by means of the program GraphPad Prism 4 (GaphPad Software, La Jolla, CA USA). In all cases, *p* < 0.05 was assumed to indicate significant differences.

## 4. Conclusions

In conclusion, using RAW264.7 macrophages as a model system, we identified the unsaturated fatty acids LNA, EPA, DHA, LA and AA to affect macrophage function, thereby driving the immune defense in an anti-inflammatory direction. The concentration of free fatty acids tested in our system match physiological conditions [36] thus underlining the relevance of the results obtained. Our data provide first evidence that PUFA from both the *n*-3 and the *n*-6 family down-regulate the synthesis of: (*i*) the pro-inflammatory cytokines IL-1β, IL-6 and TNF-α; (*ii*) the co-stimulatory molecule CD86; as well as (*iii*) the antimicrobial polypeptide lysozyme. Interestingly, the action of the fatty acids partially depended on the activation status of the macrophages. It is particularly important to note that the immune-suppressive action of PUFA could also be demonstrated in case of infection of RAW264.7 with viable microorganisms of the genera *R. equi* and *P. aeruginosa*. Taken together, our data provide strong evidence that *n*-3 as well as *n*-6 fatty acids level down inflammation processes in context of chronic infections caused by persistent pathogens.

## References

[1] Stillwell W., Wassall S.R. (2003). Docosahexaenoic acid: Membrane properties of a unique fatty acid. Chem. Phys. Lipids.

[2] Wassall S.R., Stillwell W. (2008). Docosahexaenoic acid domains: The ultimate non-raft membrane domain. Chem. Phys. Lipids.

[3] Chapkin R.S., Kim W., Lupton J.R., McMurray D.N. (2009). Dietary docosahexaenoic and eicosapentaenoic acid: Emerging mediators of inflammation. Prostaglandins Leukot Essent. Fat. Acids.

[4] Galli C., Calder P.C. (2009). Effects of fat and fatty acid intake on inflammatory and immune responses: A critical review. Ann. Nutr. Metab.

[5] Walloschke B., Fuhrmann H., Schumann J. (2010). Enrichment of RAW264.7 macrophages with essential 18-carbon fatty acids affects both respiratory burst and production of immune modulating cytokines. J. Nutr. Biochem.

[6] Schumann J., Fuhrmann H. (2010). Impairment of NFkappaB activity by unsaturated fatty acids. Int. Immunopharmacol.

[7] Schmutzler S., Bachmann L., Fuhrmann H., Schumann J. (2010). PUFA-dependent alteration of oxidative parameters of a canine mastocytoma cell line. Acta Vet. Hung.

[8] Groves E., Dart A.E., Covarelli V., Caron E. (2008). Molecular mechanisms of phagocytic uptake in mammalian cells. Cell. Mol. Life Sci.

[9] Bryant C., Fitzgerald K.A. (2009). Molecular mechanisms involved in inflammasome activation. Trends Cell Biol.

[10] Russell D.G., Vanderven B.C., Glennie S., Mwandumba H., Heyderman R.S. (2009). The macrophage marches on its phagosome: Dynamic assays of phagosome function. Nat. Rev. Immunol.

[11] Hamilton T.A., Ohmori Y., Tebo J.M., Kishore R. (1999). Regulation of macrophage gene expression by pro- and anti-inflammatory cytokines. Pathobiology.

[12] Trautmann M., Lepper P.M., Haller M. (2005). Ecology of *Pseudomonas aeruginosa* in the intensive care unit and the evolving role of water outlets as a reservoir of the organism. Am. J. Infect. Control.

[13] Prescott J.F. (1991). *Rhodococcus equi*: An animal and human pathogen. Clin. Microbiol. Rev.

[14] Kamboj M., Kalra A., Kak V. (2005). *Rhodococcus equi* brain abscess in a patient without HIV. J. Clin. Pathol.

[15] Bell K.S., Philp J.C., Aw D.W., Christofi N. (1998). The genus *Rhodococcus*. J. Appl. Microbiol.

[16] Fernandez-Mora E., Polidori M., Luhrmann A., Schaible U.E., Haas A. (2005). Maturation of *Rhodococcus equi*-containing vacuoles is arrested after completion of the early endosome stage. Traffic.

[17] von B.K., Haas A. (2009). Molecular and infection biology of the horse pathogen *Rhodococcus equi*. FEMS Microbiol. Rev.

[18] Blanc D.S., Petignat C., Janin B., Bille J., Francioli P. (1998). Frequency and molecular diversity of *Pseudomonas aeruginosa* upon admission and during hospitalization: A prospective epidemiologic study. Clin. Microbiol. Infect.

[19] Strateva T., Yordanov D. (2009). *Pseudomonas aeruginosa*—A phenomenon of bacterial resistance. J. Med. Microbiol.

[20] Pechere J.C., Kohler T. (1999). Patterns and modes of beta-lactam resistance in *Pseudomonas aeruginosa*. Clin. Microbiol. Infect.

[21] Boyen F., Eeckhaut V., Van I.F., Pasmans F., Ducatelle R., Haesebrouck F. (2009). Quorum sensing in veterinary pathogens: Mechanisms, clinical importance and future perspectives. Vet. Microbiol.

[22] Veesenmeyer J.L., Hauser A.R., Lisboa T., Rello J. (2009). *Pseudomonas aeruginosa* virulence and therapy: Evolving translational strategies. Crit. Care Med.

[23] Darrah P.A., Monaco M.C., Jain S., Hondalus M.K., Golenbock D.T., Mosser D.M. (2004). Innate immune responses to *Rhodococcus equi*. J. Immunol.

[24] Weldon S.M., Mullen A.C., Loscher C.E., Hurley L.A., Roche H.M. (2007). Docosahexaenoic acid induces an anti-inflammatory profile in lipopolysaccharide-stimulated human THP-1 macrophages more effectively than eicosapentaenoic acid. J. Nutr. Biochem.

[25] Mullen A., Loscher C.E., Roche H.M. (2010). Anti-inflammatory effects of EPA and DHA are dependent upon time and dose-response elements associated with LPS stimulation in THP-1-derived macrophages. J. Nutr. Biochem.

[26] Oh D.Y., Talukdar S., Bae E.J., Imamura T., Morinaga H., Fan W., Li P., Lu W.J., Watkins S.M., Olefsky J.M. (2010). GPR120 is an omega-3 fatty acid receptor mediating potent anti-inflammatory and insulin-sensitizing effects. Cell.

[27] Mitchell J.A., Paul-Clark M.J., Clarke G.W., McMaster S.K., Cartwright N. (2007). Critical role of toll-like receptors and nucleotide oligomerisation domain in the regulation of health and disease. J. Endocrinol.

[28] Kumar H., Kawai T., Akira S. (2011). Pathogen recognition by the innate immune system. Int. Rev. Immunol.

[29] Park J.H., Kim Y.G., Nunez G. (2009). RICK promotes inflammation and lethality after Gram-negative bacterial infection in mice stimulated with lipopolysaccharide. Infect. Immun.

[30] Olsson S., Sundler R. (2006). The role of lipid rafts in LPS-induced signaling in a macrophage cell line. Mol. Immunol.

[31] Wong S.W., Kwon M.J., Choi A.M., Kim H.P., Nakahira K., Hwang D.H. (2009). Fatty acids modulate Toll-like receptor 4 activation through regulation of receptor dimerization and recruitment into lipid rafts in a reactive oxygen species-dependent manner. J. Biol. Chem.

[32] Bishop-Bailey D., Bystrom J. (2009). Emerging roles of peroxisome proliferator-activated receptor-beta/delta in inflammation. Pharmacol. Ther.

[33] Lee J.Y., Ye J., Gao Z., Youn H.S., Lee W.H., Zhao L., Sizemore N., Hwang D.H. (2003). Reciprocal modulation of Toll-like receptor-4 signaling pathways involving MyD88 and phosphatidylinositol 3-kinase/AKT by saturated and polyunsaturated fatty acids. J. Biol. Chem.

[34] Martinez J.G., Waldon M., Huang Q., Alvarez S., Oren A., Sandoval N., Du M., Zhou F., Zenz A., Lohner K. (2009). Membrane-targeted synergistic activity of docosahexaenoic acid and lysozyme against *Pseudomonas aeruginosa*. Biochem. J.

[35] Helal R., Melzig M.F. (2010). New aspects in the synthesis and secretion of lysozyme by cultured human monocyte cell lines. In Vitro. Cell. Dev. Biol. Anim.

[36] Leaf D.A., Connor W.E., Barstad L., Sexton G. (1995). Incorporation of dietary *n*-3 fatty acids into the fatty acids of human adipose tissue and plasma lipid classes. Am. J. Clin. Nutr.

